# Low serum free IL-18 is a novel potential marker for predicting infectious events in patients at dialysis initiation

**DOI:** 10.1093/ckj/sfaf094

**Published:** 2025-04-09

**Authors:** Takashi Tawara-Iida, Joichi Usui, Itaru Ebihara, Takashi Ishizu, Masaki Kobayashi, Yoshitaka Maeda, Hiroaki Kobayashi, Kunihiro Yamagata, the Study Group of the Ibaraki Dialysis Initiation Cohort Study, Hideko Nakamura, Kenji Takada, Koichi Kozaki, Satoshi Iwabuchi, Tadashi Iitsuka, Kenta Nishiki, Hideaki Takasaki, Takashi Takita, Masami Nakajima, Sumiko Honma, Youichi Akai, Genzou Ishizuka, Koichi Issiki, Takako Saito, Hitoshi Iwamoto, Akira Ohishi, Masakazu Ohtsuka, Atsushi Ono, Hidehiko Kashiwabara, Takuro Kanekawa, Naoaki Kanamori, Fumika Kaneda, Hiroshi Kikuchi, Masashi Kubo, Hiromi Kurosawa, Takeshi Shiraishi, Tatsuo Shiigai, Masayoshi Shima, Tokuo Takahashi, Hideki Matsukawa, Minoru Tokoi, Sadao Tsunematsu, Atsushi Tsuruta, Masao Deguchi, Masahiro Hayakawa, Makoto Hiroi, Nobuki Maeda, Takanobu Hoshino, Tetsu Yamaguchi, Kota Yamada, Takashi Ishizu, Atsushi Takeda, Ikuo Takahashi, Takamichi Yuhara, Tadashi Kondo, Syoji Ooba, Yasunobu Ogura, Hisaya Tachibana, Hiroshi Ookawa, Toshihiro Fujii

**Affiliations:** Department of Nephrology, Institute of Medicine, University of Tsukuba, Tsukuba, Japan; Department of Nephrology, Institute of Medicine, University of Tsukuba, Tsukuba, Japan; Department of Nephrology, Mito Saiseikai General Hospital, Mito, Japan; Department of Renal and Dialysis Medicine, Tsukuba Central Hospital, Ushiku, Japan; Central Jin Clinic, Ryugasaki, Japan; Department of Nephrology, Tokyo Medical University Ibaraki Medical Center, Ami, Japan; Nephrology Division, Department of Internal Medicine, JA Toride Medical Center, Toride, Japan; Department of Nephrology, Ibaraki Prefectural Central Hospital, Kasama, Japan; Department of Nephrology, Institute of Medicine, University of Tsukuba, Tsukuba, Japan

**Keywords:** dialysis initiation, free IL-18, infection, multicenter prospective cohort study

## Abstract

**Background:**

Compared to the general population, individuals who are undergoing hemodialysis are at a higher risk of contracting severe infectious diseases, and their mortality rate from infectious diseases is also higher. We investigated the serum free interleukin-18 [free state of interleukin-18 (IL-18)] concentration as a prognostic factor for hemodialysis patients' infection risk.

**Methods:**

The Ibaraki Dialysis Initiation Cohort (iDIC) study is a multicenter prospective cohort investigation of patients undergoing a new initiation of dialysis in a local region of Japan. We performed a survival analysis of several events requiring hospitalization and compared the Kaplan–Meier curves of the “low” and “high” serum free IL-18 concentration groups. To adjust for confounding factors, we also performed a Cox proportional hazards analysis.

**Results:**

We analyzed the serum free IL-18 concentration of samples from 295 patients randomly selected from the blood sample bank of the iDIC study. The mean free IL-18 concentration was 8.7 ± 5.3 pmol/l. The cumulative incidence of infectious events was significantly higher in the low free IL-18 group (<6.0 pmol/l, log-rank test *P *< .01). The Cox proportional hazards analysis revealed that low serum free IL-18 (<6.0 pmol/l) was an independent factor associated with the development of infectious events. Total IL-18 and IL-18BP (binding protein) showed no association with infectious events.

**Conclusion:**

A low serum free IL-18 concentration in the dialysis initiation period is a potential marker for predicting the development of severe infection in these patients.

KEY LEARNING POINTS **What was known:**

- The life prognosis of dialysis patients is generally poor, and it is important to improve the infections that are associated with dialysis patients’ prognoses. Identifying patients on dialysis who are at high risk of developing a severe infection could help prevent the development of infections.
- IL-18 has an important role in immune responses in infectious diseases. Free IL-18 is important for the evaluation of IL-18 activity and is a potential prognostic marker for infection in dialysis patients, but this has not been established.

**This study adds:**

- We investigated the serum free IL-18 level as a predictor of the development of severe infections requiring hospitalization.
- Low (<6.0 pmol/l) serum free IL-18 at the time of dialysis induction was associated with the development of infectious events.

**Potential impact:**

- Patients with low serum free IL-18 at the time of dialysis induction are at high risk for infectious diseases.
- Active infection management may also lead to improved prognoses for patients at high risk for infectious diseases who are identified by their serum free IL-18 concentration.

## INTRODUCTION

Despite medical advances, the mortality rate among individuals who are undergoing dialysis remains higher than that of the general population, and it is thus an important challenge to improve the life prognoses of dialysis patients. Infectious diseases in particular account for 10.0% of the deaths of dialysis patients and are the second most common cause of hospitalizations of dialysis patients in the USA [1]. The prevention, early diagnosis, and appropriate treatment of infectious diseases are therefore critical for improving the prognoses of dialysis patients, and the identification of patients on dialysis who are at high risk of developing severe infection could help prevent the development of infections and improve the patients’ prognoses.

Several predictors of the development of infection and prognosis in dialysis and renal transplant patients have been reported [2–4, 5]. In the present study, we focused on free interleukin-18 (IL-18)—a free state of IL-18 that is known to be associated with the development of infection in other diseases—as a factor for predicting the risk of infection in dialysis patients. Discovered by Okamura *et al.* in 1995 [6], IL-18 is an 18.4-kDa cytokine and a member of the interleukin-1 superfamily. It induces a production of interferon-gamma (IFN-γ), which is involved in the Th1-type immune response. IL-18 also plays an important role in the host defense against pathogenic microorganisms [7–13, 14].

IL-18 has been shown to be associated with a variety of clinical conditions, and it has an important role in immune responses in infectious diseases [15, 16, 17]. IL-18 binding protein (IL-18BP) binds to IL-18 and inhibits the activity of IL-18 [18]. Free IL-18, which does not bind IL-18BP and is able to bind to the IL-18 receptor, is important for the evaluation of IL-18 activity [19]. Clinical case reports also recommend evaluations of serum free IL-18 [20–22]. The precise serum free IL-18 levels of dialysis patients have not been established [23, 24].

Against this research background, we decided to investigate the serum free IL-18 level as a predictor of both the development of severe infection requiring hospitalization and the prognosis in dialysis patients. We used the Ibaraki Dialysis Initiation Cohort (iDIC) study, which is a prospective cohort study enrolling dialysis-induced patients in Japan, to examine the associations between serum free IL-18 values at the time of dialysis initiation and clinical findings at the time of dialysis initiation and the development of severe infection during maintenance dialysis.

## MATERIALS AND METHODS

### Patient population

Our analyses were based on the iDIC study of new-induction dialysis patients in Ibaraki Prefecture, Japan [25]. The iDIC study included patients who were newly introduced to dialysis during the 3-year period from January 2013 to December 2015 and were on maintenance dialysis at one of 60 participating centers in Ibaraki Prefecture. A total of 636 patients were enrolled in the iDIC study.

The primary endpoint of the iDIC study was the mortality rate at 2 years after dialysis induction, and the secondary endpoint was the incidence of hospitalization events due to infection, malignancy, cardiovascular disease, peripheral vascular disease, and other causes until 2 years after dialysis induction. The infections included pulmonary infections (e.g. viral pneumonia, bacterial pneumonia, influenza, abscess of lung), genitourinary infections (e.g. pyelonephritis), gastrointestinal infections (e.g. appendicitis, diverticulitis, cholecystitis, cholangitis, peritonitis), peritonitis, soft-tissue infections (e.g. cellulitis), joint or bone infections (e.g. infective arthritis), and endocarditis. A unique feature of the iDIC study is that serum and urine samples from registered patients at the time of dialysis induction were collected and banked. All blood and urine samples were taken before the patients’ first dialysis sessions. In the present study, we used 295 specimens that we randomly selected from among the 531 serum specimens in the iDIC study bank. We performed simple random sampling and extracted 295 samples from 531 samples.

### Measurement of free IL-18

The serum total IL-18 concentration and the serum IL-18BP concentration were measured from the patient's serum at the time of dialysis induction. IL-18 and IL-18BP are in a constant state of equilibrium with free IL-18, free IL-18BP, and the IL-18/IL-18BP complex, and they exist in a constant ratio under the law of mass action. The measured serum total IL-18 concentration represents the sum of the serum IL-18 which forms the IL-18–IL-18BP complex plus serum free IL-18 [19]. The serum total IL-18 concentration and the serum IL-18BP concentration were measured using an IL-18 enzyme-linked immunosorbent assay (ELISA) kit (MBL, Tokyo) and an IL-18BP ELISA kit (R&D Systems, Minneapolis, MN, USA). The measurements were performed according to the manual accompanying each kit. Samples were assayed in duplicate, and the mean value of the obtained values was used as the assay value.

Using the total IL-18 concentration and serum IL-18BP concentration of the respective patient, we calculated the serum free IL-18 concentration according to the law of mass action as described [20, 26]:


\begin{eqnarray*}
Kd = \frac{{\left[ {{\mathrm{free\ IL}} - 18} \right]{\lceil\mathrm{free}\ IL} - 18{\mathrm{\ BP\rceil}}}}{{\left[ {{\mathrm{IL}} - 18 \cdot {\mathrm{IL}} - 18{\mathrm{\ BPcomplex}}} \right]}}
\end{eqnarray*}

\begin{eqnarray*}
\because \left[ {{\mathrm{IL}} - 18} \right] = \left[ {{\mathrm{free\ IL}} - 18} \right] + \left[ {{\mathrm{IL}} - 18 \cdot {\mathrm{IL}} - 18{\mathrm{\ BPcomplex}}} \right]
\end{eqnarray*}

\begin{eqnarray*}
\because \left[ {{\mathrm{IL}} - 18{\mathrm{\ BP}}} \right] = \left[ {\mathrm{free}{\mathrm{\ }}\rm IL - 18{\mathrm{\ }}\rm BP} \right] + \left[ \rm {IL - 18 \cdot {\rm IL} - 18{\mathrm{\ }}\mathrm{BPcomplex}} \right]
\end{eqnarray*}

\begin{eqnarray*}
\left[ {{\mathrm{free\ IL}} - 18} \right] = \frac{{ - \left\{ {\left[ {{\mathrm{IL}} - 18{\mathrm{BP}}} \right] - \left[ {{\mathrm{IL}} - 18} \right] + K{\mathrm{d}}} \right\} + \sqrt {{{\left\{ {\left[ {{\mathrm{IL}} - 18{\mathrm{BP}}} \right] - \left[ {{\mathrm{IL}} - 18} \right] + K{\mathrm{d}}} \right\}}}^2 + 4K{\mathrm{d}}\left[ {{\mathrm{IL}} - 18} \right]} }}{2}
\end{eqnarray*}

where [free IL-18] is the serum free IL-18 concentration (mol/l); [free IL-18BP] is the serum free IL-18BP concentration (mol/l); [IL-18] is the serum total IL-18 concentration (mol/l); [IL-18BP] is the serum IL-18BP concentration (mol/l); [IL-18BP] is the serum IL-18BP concentration (mol/l); [IL-18 18 · IL-18BP complex] is the serum IL-18 and IL-18 complex concentration (mol/l); and *K*_d_ is the dissociation constant. We set the dissociation constant *K*_d_ to 0.4 nM, referring to a previous report [19].

### Data collection

We collected the patients’ clinical data at the time of dialysis induction in the high and low serum free IL-18 groups, including age, gender, history of renal biopsy, diabetes, hypertension, medical history (myocardial infarction, stroke, peripheral vascular disease, malignancy), family history of renal disease, underlying renal disease, body mass index (BMI), blood pressure, hemoglobin, urea nitrogen, serum creatinine, estimated glomerular filtration rate (eGFR), serum uric acid, serum albumin, serum total cholesterol, serum corrected calcium, serum phosphorus, hemoglobin A1c (HbA1c), intact parathyroid hormone (PTH), urinary protein, microhematuria, and serum C-reactive protein (CRP). We also compared the two groups’ cumulative incidence of infectious events.

### Statistical analyses

Receiver operating characteristic (ROC) curves were used to predict infectious events. The cut-off value of the serum free IL-18 concentration to maximize the area under the curve (AUC) was established with reference to the Youden index. Then, based on the cut-off value, we divided the patients into a low serum free IL-18 concentration group and a high serum free IL-18 concentration group and compared the groups' clinical data.

Qualitative data are presented as the number of cases and percentage (%), and quantitative data are presented as the mean and standard deviation (SD). The *χ*^2^ test was used to compare qualitative data between the two groups. The Mann–Whitney *U*-test was used to compare quantitative data. A survival analysis was performed for the cumulative incidence of infectious events, and Kaplan–Meier curves were drawn using the log-rank test for the comparison of Kaplan–Meier curves between the low and high serum free IL-18 concentration groups. A Cox proportional hazards analysis was performed using infection as a dependent variable and low free IL-18 as an independent variable.

We also included the variables that have been reported to be associated with infection as independent variables [2, 3]. There was no multicollinearity among the independent variables of the Cox proportional hazards analysis.

We analyzed the relationship between infectious events and total IL-18 and IL-18BP as well as free IL-18 using Kaplan–Meier curves and a Cox proportional hazards analysis. The significance level was set at *P *= .05. IBM SPSS Statistics v.25.0 (IBM, Armonk, NY, USA) was used for the statistical analyses.

### Ethical considerations

Each patient's informed consent for participation in this study was obtained. The collected data were consolidated and anonymized at each institution and submitted to the University of Tsukuba Secretariat for registration. This study was approved by the Ethics Review Committee of the University of Tsukuba Hospital (approval no. H24-116) and was conducted in accord with the Ethical Guidelines for Epidemiological Research June 2002 (partially revised in December 2008) of the Ministry of Education, Culture, Sports, Science and Technology and the Ministry of Health, Labor and Welfare, Japan.

## RESULTS

This study is a secondary analysis of the iDIC study. A total of 295 patients (195 males and 100 females) were randomly selected from among the patients enrolled in the iDIC study. The clinical findings of the eligible patients at the time of dialysis induction are summarized in Table 1: 276 patients were on hemodialysis, and the other 19 patients were on peritoneal dialysis. The median follow-up period of the analyzed patients was 730 days (range 1–730 days). The patients’ mean age was 67.5 ± 12.5 years, their mean serum creatinine was 9.1 ± 2.9 mg/dl, the mean eGFR was 5.6 ± 1.9 ml/min/1.73 m^2^, and the mean serum CRP was 2.0 ± 4.9 mg/dl. Among all 295 patients, the mean serum free IL-18 concentration was 8.7 ± 5.3 pmol/l, the mean serum total IL-18 concentration was 29.9 ± 18.7 pmol/l, and the mean serum IL-18BP concentration was 1034.0 ± 468.2 pmol/l.

**Table 1.** Clinical findings at baseline in the low and high serum free IL-18 groups.

	Free IL-18<6.0 pmol/l	Free IL-18≧6.0 pmol/l	Total entries	*P* value
Number	83	212	295	
(PD/HD)	77/6	199/13	276/19	.51
age (years)	69.4 ± 11.1	66.8 ± 12.9	67.5 ± 12.5	.13
gender (male/female)	54/29	141/71	195/100	.81
renal biopsy (*n*, %)	13, 15.7%	28, 13.2%	41, 14.9%	.61
diabetes at dialysis initiation (*n*, %)	47, 56.6%	121, 57.1%	168, 57.3%	.79
hypertension at dialysis initiation (*n*, %)	75, 90.4%	202, 95.3%	277, 94.9%	.18
prior myocardial infarction (*n*, %)	9, 10.8%	21, 9.9%	30, 11.8%	.54
prior stroke (*n*, %)	14, 16.9%	31, 14.6%	45, 17.2%	.65
prior peripheral vascular disease (*n*, %)	12, 14.5%	12, 5.7%	24, 10.5%	.03
prior malignancy (*n*, %)	14, 16.9%	26, 12.3%	40, 15.4%	.57
family history of kidney disease (*n*, %)	3, 3.6%	16, 7.5%	19, 9.1%	.18
primary kidney disease (*n*, %)				
diabetic nephropathy	40, 48.2%	104, 49.1%	144, 48.8%	.97
hypertensive nephrosclerosis	14, 16.9%	40, 18.9%	54, 18.3%	
chronic glomerulonephritis	16, 19.3%	42, 19.8%	58, 19.7%	
autosomal dominant polycystic kidney disease	1, 1.2%	3, 1.4%	4, 1.4%	
rapidly progressive glomerulonephritis	4, 4.8%	7, 3.3%	11, 3.7%	
other disease	5, 6.0%	12, 5.7%	11, 5.8%	
unknown cause	3, 3.6%	4, 1.9%	7, 2.4%	
BMI (kg/m^2^)	24.0 ± 4.3	24.1 ± 4.0	24.1 ± 4.1	.89
systolic blood pressure (mmHg)	146.7 ± 27.2	151.3 ± 25.3	150.0 ± 25.9	.12
diastolic blood pressure (mmHg)	75.4 ± 17.0	78.9 ± 16.5	77.9 ± 16.7	.07
hemoglobin (g/dl)	8.8 ± 1.7	9.0 ± 2.0	8.9 ± 1.9	.96
blood urea nitrogen (mg/dl)	94 ± 33.9	96.1 ± 29.4	95.5 ± 30.7	.45
serum creatinine (mg/dl)	9.0 ± 2.9	9.1 ± 2.9	9.1 ± 2.9	.60
eGFR (ml/min/1.73 m^2^)	5.6 ± 2.0	5.6 ± 1.8	5.6 ± 1.9	.93
serum uric acid (mg/dl)	8.1 ± 2.3	8.2 ± 2.3	8.2 ± 2.3	.92
serum albumin (g/dl)	3.4 ± 0.7	3.3 ± 0.7	3.3 ± 0.7	.53
serum total cholesterol (mg/dl)	161.5 ± 64.6	158.5 ± 47.3	159.3 ± 52.4	.63
serum corrected calcium (mg/dl)	8.6 ± 0.9	8.5 ± 1.1	8.5 ± 1.1	.42
serum phosphate (mg/dl)	6.2 ± 1.9	5.8 ± 0.8	6.3 ± 1.9	.78
hemoglobin A1c (%)	6.1 ± 1.2	5.8 ± 0.8	5.8 ± 0.9	.20
intact PTH (pg/ml)	299 ± 241.1	282.7 ± 207.6	288.1 ± 217.8	.89
urinary protein (g/day or g/gCr)	3.8 ± 3.6	3.6 ± 3.3	3.7 ± 3.4	.91
microhematuria (*n*, %)	44, 53.0%	125, 59.0%	169, 57.3%	.65
serum CRP (mg/dl)	2.4 ± 5.9	1.9 ± 4.4	2.0 ± 4.9	.17
cumulative death (*n*, %)	15, 18.1%	25, 11.8%	40, 13.6%	.16
cumulative incidence of hospitalization				
cardiovascular disease (*n*, %)	7, 8.4%	25, 11.8%	32, 10.8%	.40
malignancy (*n*, %)	4, 4.8%	8, 3.8%	12, 4.1%	.69
infection (*n*, %)	12, 14.5%	8, 3.8%	20, 6.8%	<.01
peripheral vascular disease (*n*, %)	2, 2.4%	3, 1.4%	5, 1.7%	.55
other causes (*n*, %)	20, 24.1%	47, 22.2%	67, 22.7%	.72

For each 2-year prognosis interval, the cumulative mortality rate after the induction of dialysis was 40 patients (13.6%); the cumulative incidence of hospitalizations related to cardiovascular disease was 32 patients (10.8%). The cumulative incidence of hospitalizations related to malignancy was 12 patients (4.1%); the cumulative incidence of hospitalizations related to infectious disease was 20 patients (6.8%); and the cumulative incidence of hospitalizations related to peripheral vascular disease was five patients (1.7%). With regard to hospitalization events due to infections, the type of infection and the infectious organs are as follows. A total of 20 patients developed a severe infection requiring hospitalization: seven with respiratory infections five with abdominal infections two with pleurisy one patient each with a renal abscess, infective endocarditis, and mandibulitis and three patients developed sepsis with unknown causative organ.

**Figure 1.**
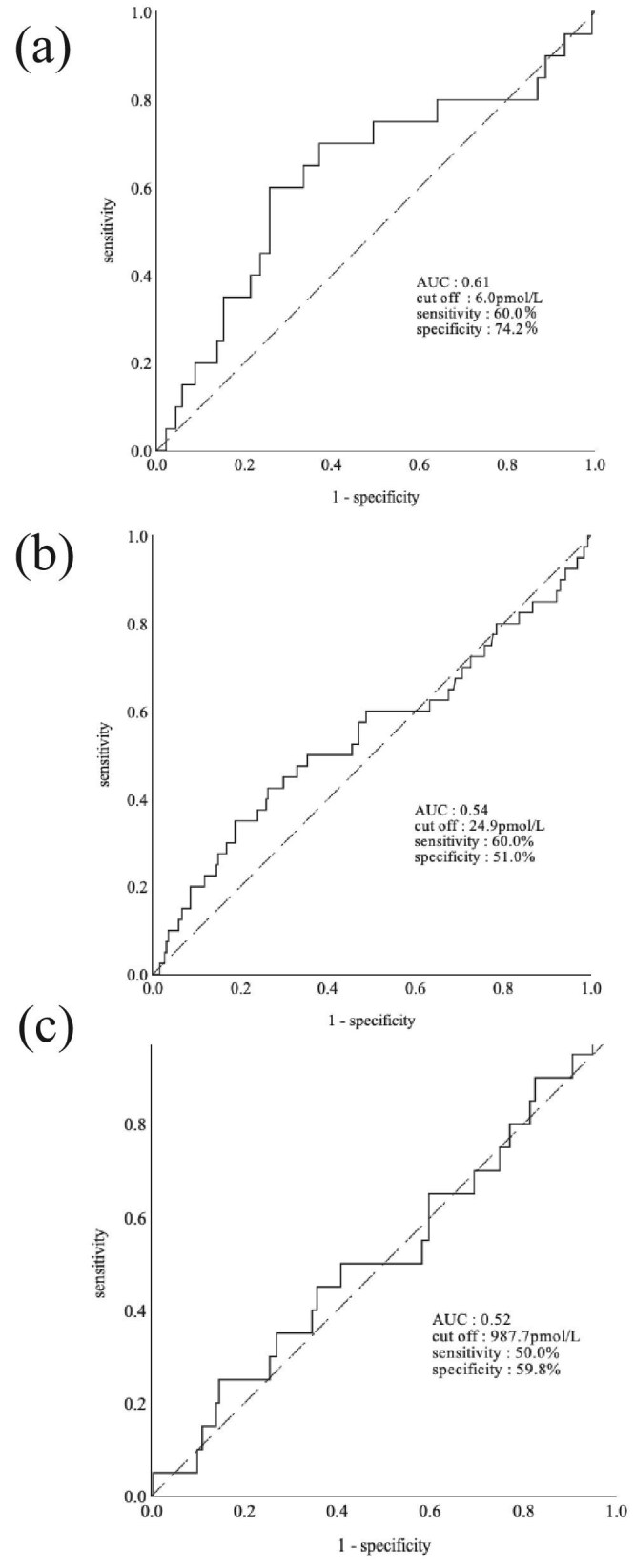
The ROC curve for free IL-18 concentrations in relation to the risk of developing infection. (**a**) The AUC of the ROC curve was 0.61. The sensitivity was 60.0% and the specificity was 74.2%. The ROC curves for the patients’ total IL-18 (**b**) and IL-18BP (**c**) concentrations in relation to the risk of developing infection. The AUCs were 0.54 and 0.52, respectively.

To select the optimal serum IL-18 endpoint as a clinical indicator, we examined the association between three measures—the patients' serum free IL-18, total IL-18, and IL-18BP concentrations—and infectious disease events. Figure 1a provides the ROC curve of the serum free IL-18 concentrations for the onset of hospitalization due to infectious events. The AUC of the ROC curve was 0.61. The serum free IL-18 concentration corresponding to the Youden index was 6.0 pmol/l, which we used as the cut-off value. When the cut-off value was 6.0 pmol/l, the sensitivity and specificity for predicting the onset of hospitalization due to infectious events were 60.0% and 74.2%, respectively. The AUCs of the ROC curves for the serum total IL-18 and serum IL-18BP concentrations were 0.54 and 0.52, respectively (Fig. 1b, c), which are lower than the AUC of the serum free IL-18 concentration.

**Figure 2.**
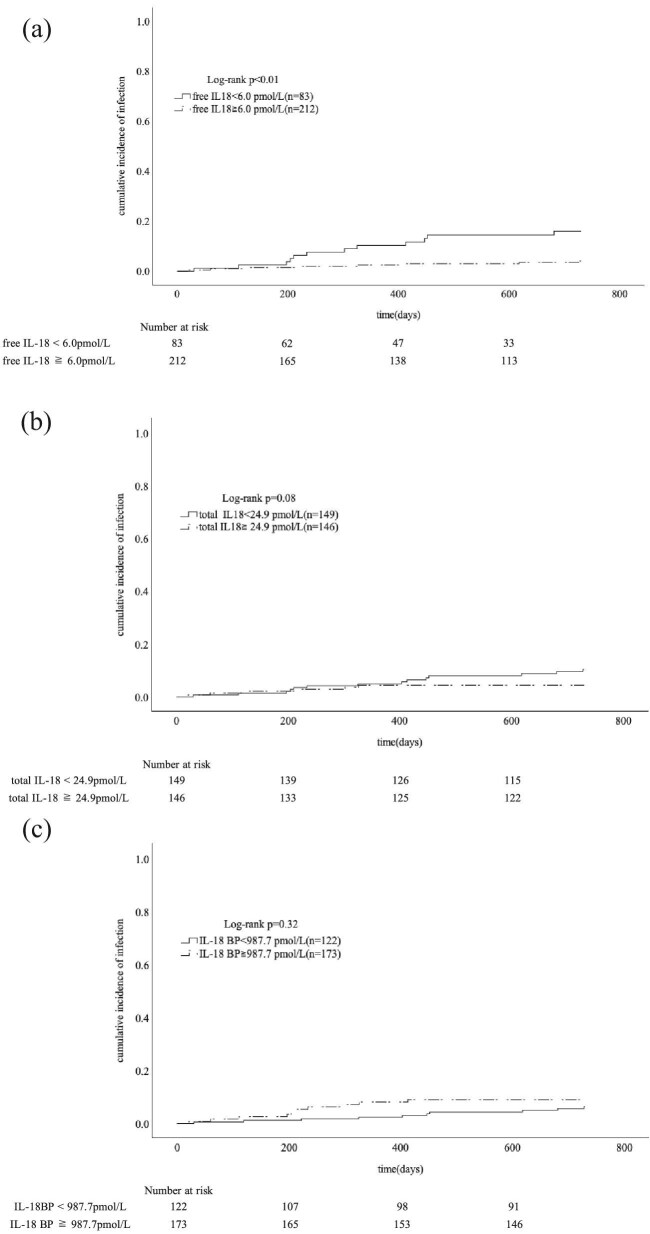
The cumulative incidence of infection in the high and low free IL-18 groups. (**a**) The cut-off value was 6.0 pmol/l. The cumulative incidence of infectious disease events was significantly higher in the low serum free IL-18 group (log-rank test, *P *< .01). The cumulative incidence of infectious disease events in the high and low total IL-18 (**b**) and IL-18BP (**c**) groups, which did not differ significantly.

Our comparison of the cumulative incidence of infectious events in the low and high serum free IL-18 concentration groups showed a significantly higher rate in the low serum free IL-18 concentration group (Fig. 2a, log-rank test: *P *< .01). There was no significant difference in the cumulative incidence of infectious events when the serum total IL-18 and IL-18BP concentrations were compared by group (Fig. 2b, c, log-rank test: *P *= .08 and .32, respectively). Based on these findings, the serum free IL-18 concentration was revealed as the optimal measure of serum IL-18 as a clinical indicator.

We next investigated the relationship between the low and high free IL-18 groups’ serum free IL-18 concentrations and their clinical findings at the time of dialysis induction (Table 1), using a serum free IL-18 concentration of 6.0 pmol/l as the cut-off value to predict the risk of infection. The analysis revealed that the likelihood of a previous peripheral vascular disease was significantly higher in the low free IL-18 concentration group. Other clinical and laboratory findings were not significantly different between the two groups. The cumulative incidence of hospitalizations related to infections after the induction of dialysis was significantly higher in the low free IL-18 concentration group. There were no significant between-group differences in mortality, the cumulative incidence of hospitalizations related to cardiovascular disease, malignancy, or peripheral vascular disease after the induction of dialysis. Although a significant difference was not observed, there was a trend of a negative relationship between free IL-18 and CRP values (data not shown). Our comparison of the serum free IL-18 concentration and each clinical parameter in a single correlation analysis revealed no significant correlation.

A Cox proportional hazards regression analysis was performed for low serum free IL-18 levels (serum free IL-18 concentration <6.0 pmol/l), with age, serum albumin, serum corrected calcium, serum phosphorus, and intact PTH used as adjustment factors (Table 2). Low serum free IL-18 levels were independently and significantly associated with the development of infectious events [hazard ratio (HR) 8.03, 95% confidence interval (CI) 1.46–44.14, *P *= .02]. We performed the same analysis for low serum total IL-18 concentrations (<24.9 pmol/l) and low serum IL-18BP concentrations (<987.7 pmol/l), and the results demonstrated that these low levels were not associated with the development of infectious events (HR 1.875, 95%CI: 0.34–10.25, *P *= .47; HR 1.25, 95%CI: 0.28–5.67, *P *= .77, respectively).

**Table 2.** COX proportional hazards analysis with infection as the dependent variable and low serum free IL-18 as the independent variable.

		95.0% confidence interval of hazard ratio		
Independent variable	Hazard ratio	Lower limit	Upper limit	*P* value
serum free IL-18 <6.0 pmol/l	8.03	1.46	44.14	.02
eGFR	1.24	0.65	2.37	.51
serum CRP	0.9	0.71	1.13	.36
age	0.99	0.93	1.05	.68
serum albumin	1.37	0.2	9.23	.74
hemoglobin	1.3	0.66	2.56	.45
serum corrected calcium	2.23	0.78	6.38	.14
serum phosphate	1.16	0.68	1.97	.59
intact PTH	1	1	1	.33

## DISCUSSION

We measured three indicators related to IL-18, i.e. the serum IL-18, IL-18BP, and calculated serum free IL-18, in dialysis patients from the iDIC study and examined the association between these concentrations and infectious events. This is the first report of associations between serum free IL-18 and severe infectious events requiring hospitalization in dialysis patients. Our analyses revealed that a low serum free IL-18 concentration at the time of dialysis induction was associated with the development of future infections. The significance and importance of the serum free IL-18 concentration evaluated in this study are discussed in detail next, along with the dynamics of the serum free IL-18 concentration in patients with renal failure (with reference to previous reports on the effects of renal dysfunction and uremia).

We compared the means of the serum total IL-18, serum IL-18 BP, and serum free IL-18 concentrations in the present patients with those reported in healthy participants, patients with chronic renal failure, dialysis patients, patients with sepsis, and patients with various diseases (Table 3) [20, 21, 27–29, 30]. The mean serum free IL-18 concentrations of the chronic renal failure patients and dialysis patients were higher than those of the healthy subjects [27]. It was reported that high serum free IL-18 concentrations were correlated with a low eGFR in patients with chronic renal failure [31]. The serum IL-18 concentration was also reported to be positively correlated with the urinary β2 microglobulin levels, and it was suggested that a high serum IL-18 concentration may be associated with tubulointerstitial damage [32]. We speculate that the serum IL-18 concentration may be affected by reduced clearance due to reduced glomerular filtration and reduced reabsorption from the tubular interstitium. The serum free IL-18 concentrations at the time of dialysis induction in this study's patients were higher than the reported values in chronic renal failure patients and dialysis patients, and it can be inferred that this concentration reflects the effects of advanced renal failure during the induction phase of dialysis.

**Table 3.** Comparison of serum IL-18, IL-18 BP, and free IL-18 concentration by disease.

	*N*	Total IL-18 (pmol/l)	IL-18 BP (pmol/l)	Free IL-18 (pmol/l)
Healthy [27]	29	1.0 ± 0.2	193.2 ± 22.7	0.7 ± 0.2
Chronic renal failure [27]	29	7.7 ± 0.4	426.1 ± 39.8	1.3 ± 0.2
Hemodialysis [27]	40	5.1 ± 0.7	744.3 ± 45.5	2.2 ± 0.3
Sepsis [20]	42	13.6–543.5	1278.4	13.6–163.0
Ulcerative colitis [28]	93	21.4	267	13.6
Crohn's disease [28]	135	29.7	284.1	18.5
Wegener's disease [29]	8	13	823.9	4.6
SLE [21]	48	21.7	852.3	9.1
Coronary artery disease [30]	196	19.3	778.4	6.8
Dialysis initiation period (this study)	295	29.9 ± 18.7	1034.0 ± 468.2	8.7 ± 5.3

With regard to the elevated serum free IL-18 concentration in patients with renal failure, it is necessary to consider the effect of uremia. Patients with chronic renal failure have been shown to have increased secretions of proinflammatory cytokines and thereby increased oxidative stress and ultimately a chronic hyperinflammatory state, with the cells responsible for the innate immune system being permanently stimulated [33]. This persistent immune-stimulated state under uremia may contribute to the high serum free IL-18 concentration in patients with renal failure. In particular, the dialysis initiation phase, which was the focus of the present study, produces a more highly uremic environment than the chronic renal failure phase or hemodialysis phase, and this is consistent with our present finding that the serum free IL-18 concentrations are higher during the introduction of dialysis than during the chronic renal failure phase or hemodialysis phase.

Our analyses showed that low (<6.0 pmol/l) serum free IL-18 at the time of dialysis induction was associated with the development of infectious events requiring hospitalization. As mentioned previously, the serum free IL-18 value <6.0 pmol/l is low among individuals who are in the induction phase of hemodialysis, but it can be high compared to healthy individuals. Based on the present data, it can be concluded that the relatively low serum free-IL18 levels during the induction phase of dialysis are associated with the development of infections.

There have been numerous reports about an association between IL-18 and infection. IL-18 is a proinflammatory cytokine that plays an important role in host defense against various pathogens. A deficiency of IL-18 can impair the immune response to infection in several ways [34]. IL-18 is a potent inducer of interferon-gamma (IFN-γ), which is critical for eliminating intracellular microbes. Low levels of IL-18 can lead to a decreased production of IFN-γ, potentially compromising the body's ability to fight certain infections. IL-18 also enhances the cytotoxic activities of natural killer (NK) cells and CD8^+^ T cells. Low levels of IL-18 could lead to a reduced activation of these important immune cells, weakening the overall immune response.

Weijer *et al*. suggested that IL-18 plays a protective role in the immune response to bacterial infection [15], and Kinoshita *et al.* reported that recombinant IL-18 enhanced the immune response in mice infected with *E. coli* [16, 17]. Low serum IFN-γ, which is induced by the production of IL-18, was reported to be a predictor of the development of infectious events in patients with end-stage renal failure [5]. Genetically predicted IL-18 has been reported to be associated with a lower risk of contracting a COVID-19 infection [35]. Collectively, these reports suggest that IL-18 plays a protective role against infection, and low IL-18 secretion is likely to confer a risk for the development of infection.

Our comparison of the average CRP levels of the low free IL-18 group with those of the high free IL-18 group revealed that the low free IL-18 group tended to have higher CRP levels. Although we did not investigate the patients’ infection status at the time of dialysis initiation, it is possible that some of patients in the low free I L-18 group developed infections that led to an increase in their CRP levels.

It has been reported that IL-37 (IL-1F7) further enhances the ability of IL-18BP to neutralize IL-18 activity by 25%–30% in human NK cell lines. This appears to be due to the formation of a complex IL-18BP–IL-37b–IL-18 receptor (IL-18R), which reduces IL-18 activity [36]. The authors of that study also reported that the addition of IL-37 reduced the production of INF-γ. The dynamics of IL-37 may be important in the context of our present investigation, but it is unclear how the formation of the complex IL-18BP–IL-37b–IL-18R affects the blood concentration of free IL-18. This will be a topic for future research.

There are several potential clinical applications of the serum free IL-18 concentration. A low serum free IL-18 concentration is a prognostic factor for the development of infectious diseases, and patients with low serum free IL-18 can be considered to be at high risk for infectious diseases. Active vaccination against infection, active nutritional management, the optimization of dialysis efficiency, the management of chronic kidney disease-mineral bone disorder, and iron management are important for the prevention of infectious diseases in dialysis patients who are at high risk for infection [37]. These active infection management approaches to infection may also lead to improved prognoses for patients at high risk for infectious diseases who are identified by their serum free IL-18 concentration.

Our analyses demonstrated no significant difference in mortality between the low and high free IL-18 groups. The causes of death were as follows: infection (*n* = 9 patients), cancer (*n* = 7), cardiovascular disease (*n* = 5), stroke (*n* = 5), heart failure (*n* = 3), renal failure (*n* = 2), gastrointestinal bleeding (*n* = 1), cirrhosis (*n* = 1), respiratory failure (*n* = 1), vasculitis (*n* = 1), and unknown (*n* = 5). Of the 40 cases of death, only nine were due to infection, which we suspect is the reason why there was no significant between-group difference in mortality.

The history of peripheral arterial disease was higher in the present low free IL-18 concentration group, but the association between peripheral vascular disease and free IL-18 was not fully evaluated in this study, because only hospitalization events for peripheral vascular disease were evaluated. The association of free IL-18 with peripheral atherosclerosis that does not lead to hospitalization is a subject for future study.

A limitation of this study is that the data were limited to data collected in the iDIC study, which may not be generalizable to other populations. In addition, the iDIC study was limited to a 2-year observation period, and the nature of our present investigation as a secondary study made it difficult to investigate longer-term prognoses. We did not evaluate the incidence of events at the time of dialysis induction, and it is difficult to assess whether these events affected serum free IL-18 levels at the time of sample collection. The primary study on which our present analyses were based, i.e. the iDIC study, did not collect information on dialysis performance (such as blood access and dialysis efficiency) or information on frailty rates and activity levels. Such information may be related to the occurrence of infectious diseases. In the iDIC study, serum and urine samples were collected only once at the time of dialysis initiation to form a sample bank, and it was difficult to assess whether blood levels of IL-18 were stable over time due to the study protocol. The situation at the time of patients’ dialysis initiation has not been studied, and the effects of infection and inflammation at the time of dialysis initiation on free IL-18 levels cannot be evaluated. Owing to the limited sample volume in the present study, additional tests such as measurements of IL-18BP-bound IL-18 and other inflammatory markers could not be performed. IL-18 may be affected by chronic inflammation in patients with chronic renal failure [33, 38], but the kinetics of IL-18 in patients under uremia with chronic renal failure are not yet clear.

In conclusion, the results of our analyses showed that in dialysis-induced patients, a low serum free IL-18 concentration at the induction phase of dialysis is a potential predictor of the development of infectious diseases. Proactive prophylactic management of infectious diseases for patients in high-risk groups identified by their serum free IL-18 concentrations may lead to improved prognoses.

## Data Availability

The data underlying this article will be shared on reasonable request to the corresponding author.

## SUPPLEMENTARY APPENDIX

Research Committee of Ibaraki Dialysis Initiation Cohort study: In addition to the authors, the following investigators and institutions participated in this study: Department of Nephrology, Ushiku Aiwa General Hospital (Dr. Hideko Nakamura); Department of Nephrology, Tsukuba Gakuen Hospital (Dr. Kenji Takada); Department of Surgery, National Hospital Organization Mito Medical Center (Dr. Koichi Kozaki); Department of Internal Medicine, Kensei General Hospital (Dr. Satoshi Iwabuchi); Department of Nephrology, Ibaraki Seinan Medical Center Hospital (Dr. Tadashi Iitsuka); Department of Nephrology, Mito Kyodo General Hospital (Dr. Kenta Nishiki); Department of Surgery, Kamisu Saiseikai Hospital (Dr. Hideaki Takasaki); Department of Internal Medicine, Mito Red Cross Hospital (Dr. Takashi Takita); Department of Nephrology, Hitachinaka General Hospital (Dr. Masami Nakajima); Division of Nephrology, Koga Red Cross Hospital (Dr. Sumiko Honma); Chikusei Kidney Clinic (Dr. Youichi Akai); Ishizuka Clinic (Dr. Genzou Ishizuka); Isshiki Clinic (Dr. Koichi Issiki); Kawashima Medical Clinic (Dr. Takako Saito); Iwamoto Clinic (Dr. Hitoshi Iwamoto); Ohishi Naika Clinic (Dr. Akira Ohishi); Tokiwa Clinic (Dr. Masakazu Ohtsuka); Mito Chuo Hospital (Dr. Atsushi Ono); Mito Chuo Clinic (Dr. Hidehiko Kashiwabara); Tajirigaoka Hospital (Dr. Takuro Kanekawa); Department of Internal Medicine, Sannoudai Hospital (Dr. Naoaki Kanamori); Medical Corporation Kamome Hitachi Clinic (Dr. Fumika Kaneda); Kikuchi Naika Clinic (Dr. Hiroshi Kikuchi); Tsukuba Jin Clinic (Dr. Masashi Kubo); Sumiyoshi Clinic Hospital (Dr. Hiromi Kurosawa); Katsuta Hospital (Dr. Takeshi Shiraishi); Shiigai Clinic (Dr. Tatsuo Shiigai); Shima Iin (Dr. Masayoshi Shima); Okubo Hospital (Dr. Tokuo Takahashi); Naka Clinic (Dr. Hideki Matsukawa); Hitachi Clinic (Dr. Minoru Tokoi); Yuki Clinic (Dr. Sadao Tsunematsu); Sakura Mito Clinic (Dr. Atsushi Tsuruta); Kitaibaraki Chuo Clinic (Dr. Masao Deguchi); Tsukuba Gakuen Clinic (Dr. Masahiro Hayakawa); Mitsukaido Sakura Hospital (Dr. Makoto Hiroi); Maeda Hospital (Dr. Nobuki Maeda); Kandatsu Hospital (Dr. Takanobu Hoshino); Yamaguchi Clinic (Dr. Tetsu Yamaguchi); Tsuchiura Beryl Clinic (Dr. Kota Yamada); Central Jin Clinic (Dr. Takashi Ishizu); Midorino Clinic (Dr. Atsushi Takeda); Moriyaekimae Clinic (Dr. Ikuo Takahashi); Department of Internal Medicine, Namegata District General Hospital (Dr. Takamichi Yuhara); Kenpoku Medical Center Takahagi Kyodo Hospital (Dr. Tadashi Kondo); Ohba Renal Clinic (Dr. Syoji Ooba); Kobuki Naika (Dr. Yasunobu Ogura); Tachibana Clinic (Dr. Hisaya Tachibana); Tonegawabashi Clinic (Dr. Hiroshi Ookawa); Fujii Hospital (Dr. Toshihiro Fujii).
